# Prognostic value of a modified systemic inflammation score in breast cancer patients who underwent neoadjuvant chemotherapy

**DOI:** 10.1186/s12885-022-10291-2

**Published:** 2022-12-02

**Authors:** Cong Jiang, Yuting Xiu, Xiao Yu, Kun Qiao, Shiyuan Zhang, Yuanxi Huang

**Affiliations:** Department of Breast Surgery, Harbin Medical University Cancer Hospital, Harbin, China

**Keywords:** mSIS, Breast cancer, Nomogram, Interaction

## Abstract

**Background and purpose:**

The modified systemic inflammation score (mSIS) system, which is constructed based on the neutrophil to lymphocyte ratio (NLR) and albumin (Alb), has not been applied to evaluate the prognosis of malignant breast cancer patients who underwent neoadjuvant chemotherapy (NAC). The present study aimed to explore the relationship between the mSIS and overall survival (OS), disease-free survival (DFS) and pathological complete response (pCR).

**Methods:**

A total of 305 malignant breast tumor patients who underwent NAC were incorporated into this retrospective analysis. We determined OS and DFS using K-M survival curves and the log-rank test. The relationship between the mSIS and OS and DFS was evaluated by a Cox regression model. A nomogram was constructed based on Cox regression analysis.

**Results:**

Patients in the mSIS low-risk group had better 5- and 8-year OS rates than those in the mSIS high-risk group (59.8% vs. 77.0%; 50.1% vs. 67.7%; X^2^ = 8.5, *P* = 0.0035, respectively). Patients in the mSIS (1 + 2 score) + pCR subgroup had the highest 5- and 8-year OS and disease-free survival (DFS) rates (OS: 55.0% vs. 75.7% vs. 84.8, 42.8% vs. 65.7% vs. 79.8%, X^2^ = 16.6, *P* = 0.00025; DFS: 38.8% vs. 54.7% vs. 76.3%, 33.3% vs. 42.3 vs. 72.1%, X2 = 12.4, *P* = 0.002, respectively). Based on the mSIS, clinical T stage and pCR results, the nomogram had better predictive ability than the clinical TNM stage, NLR and Alb.

**Conclusions:**

mSIS is a promising prognostic tool for malignant breast tumor patients who underwent NAC, and the combination of mSIS and pCR is helpful in enhancing the ability to predict a pCR.

**Supplementary Information:**

The online version contains supplementary material available at 10.1186/s12885-022-10291-2.

## Introduction

Breast cancer is the most common form of carcinoma among adult women, with the highest morbidity and mortality rates [1]. For patients with locally advanced or unresectable breast cancer, or if the tumor size to breast volume ratio is large, neoadjuvant chemotherapy (NAC) is recommended to increase the chances of performing radical surgery for unresectable cancer and breast conserving surgery for other cases, while avoiding axillary dissection [2]. Some traditional and classic biomarkers, such as human epidermal growth factor receptor-2 (HER-2), Ki-67 index, estrogen receptor (ER), and progesterone receptor (PR), have been applied to classify malignant breast tumors [3]. These biomarkers are strongly associated with the prognosis of breast carcinoma [4] and can be determined by immunohistochemistry (IHC), which is the cheapest method available for defining the breast cancer subtype (FISH and NGS sequencing are much more expensive). However, IHC can be time-consuming and complicated, so additional biomarkers are needed for breast cancer prognostication.

In recent decades, inflammation and nutritional status have been observed to be hallmarks of cancers [5, 6]. Patients with cancer experience changes in their peripheral blood due to the systemic inflammatory response, a factor that significantly affects disease progression [7]. Some hematology and nutritional parameters, such as the neutrophil to lymphocyte ratio (NLR), prognostic nutrition index (PNI) and systemic immune-inflammation index (SII), have been developed to predict the development of breast cancer [8–10].

The cutoff value is a critical value used to apply an index to a population. The Youden index, median or quartile is applied to select the optimal cutoff value, based on which patients can be divided into two groups (high and low risk). However, with these parameters, the cutoff value may be inconsistent due to varied criteria. In addition, even though a combination of different parameters can offer more comprehensive information about a single person, it increases the computational complexity. All the above problems limit the clinical utility of the defined threshold. Therefore, the development of a simple scoring system based on these inflammatory and nutritional parameters has attracted increased attention, with the goal of allowing doctors to use combinations of fewer parameters to obtain more patient information.

Recently, a novel systemic inflammation score system (SIS) based on the lymphocyte to monocyte ratio (LMR) and albumin (Alb) was found to be associated with the outcome of patients with different solid tumors [11–14], including breast cancer patients [15]. Unlike SIS, the modified systemic inflammation score system (mSIS) is a more commonly used inflammatory index combining NLR and Alb, and it has also been proven to be a predictor of the outcome of many malignant tumors [16–18]. No researchers have explored the links between the SIS or mSIS and the OS or between the DFS and pCR in breast carcinoma patients who underwent NAC. In addition, the relationships among the SIS, mSIS, traditional biomarkers and pCR is unclear. Clarifying the relationship between the mSIS and survival outcomes can help doctors make a preliminary judgment on the efficacy of NAC so that they can adjust the treatment regimens in a timely fashion.

This work explored the relationship between SIS/mSIS and the outcomes of malignant breast tumor patients who underwent NAC and studied how the mSIS, traditional biomarkers and a pathologic complete response (pCR) interact. In addition, we designed a nomogram tailored for assessing the prognosis of breast cancer patients with the mSIS. In addition, we compared its predictive value with the clinical TNM staging system.

## Patients and methods

### Patients

This study included 305 patients with breast carcinoma who underwent NAC and surgery at Harbin Medical University Cancer Hospital between February 2012 and May 2016. The inclusion criteria were as follows: (1) invasive breast cancer diagnosed by biopsy and (2) complete follow-up and clinical and pathologic data. The exclusion criteria were as follows: (1) suffering or suffering from inflammatory disease and malnutrition within 3 months prior to NAC (malnutrition [19] is diagnosed when an adult has two or more of the following conditions: insufficient energy intake [20], weight loss [21], loss of muscle mass [22], loss of subcutaneous fat [22], localized or generalized fluid accumulation [22] that may sometimes mask weight loss, and diminished functional status as measured by handgrip strength [23, 24]); and (2) distant metastasis.

This research complies with the World Medical Association Declaration of Helsinki in 1964 and subsequently amended versions. An informed consent form was signed by all of the patients before the treatment.

### Chemotherapy regimens

A clinical decision is made after IHC, with patients' preferences being considered; the NAC regimens were predominantly anthracycline- or taxane-based. AC regimen: doxorubicin (A) 60 mg/m^2^ and cyclophosphamide (C) 600 mg/m^2^; AC-T: A 60 mg/m^2^, C 600 mg/m^2^, and docetaxel (T) 90 mg/m^2^; AC-TH: A 60 mg/m^2^, C 600 mg/m^2^, T 75 mg/m^2^, and Herceptin (H) first dose 8 mg/kg, then 6 mg/kg; TAC: T 75 mg/m^2^, A 50 mg/m^2^, and C 500 mg/m^2^; EC: epirubicin (E) 100 mg/m^2^ and C 600 mg/m^2^; EC-T: E 90–100 mg/m^2^, C 600 mg/m^2^ and T 80–100 mg/m^2^. Each cycle takes 21 days. It should be noted that because of the financial burden, only some of the patients received trastuzumab, and no one received pertuzumab because it was only available in China starting in 2019.

### Classification

Breast cancer samples with 1% to 100% positive tumor nuclei were defined as ER and PR positive, while samples with < 1% or 0% positive tumor cell nuclei were defined as negative [25]. Different from past definitions, the in situ hybridization (ISH) test showing negative results with HER2 IHC scores of 1 + or 2 + was considered low expression. A HER2 IHC score of 0 was treated as HER-2 negative, and 3 + or 2 + with positive ISH was HER-2 positive [26].

Age, height, weight, lymphocytes (L), neutrophils (N), monocytes (M), hemoglobin (Hb), platelets (P), and globulin (GLOB) were converted into binary variables by the median. Patients were divided into two groups by the Chinese standard of body mass index (BMI) [27].

The SIS was calculated as follows: a patient with LMR > 2.96 and Alb > 46.3 scored 0; LMR > 2.96 or Alb > 46.3 scored 1; LMR ≤ 2.96 and Alb ≤ 46.3 scored 2. Based on the score, the patients were divided into the SIS low-risk group (scored 0) and the SIS high-risk group (scored 1 or 2).

The mSIS was calculated as follows: a patient with NLR > 2.24 and Alb ≤ 46.3 scored 0; NLR > 2.24 or Alb ≤ 46.3 scored 1; and NLR ≤ 2.24 and Alb > 46.3 scored 2. Those who scored 1 or 2 were categorized as the mSIS low-risk group, and those who scored 0 were categorized as the mSIS high-risk group.

### Follow-up

After surgery, all patients received postoperative follow-up in outpatient or inpatient care every 3 months for the first two years, every 6 months for the next three years, and annually thereafter. The follow-up lasted until December 2020 or the date of death from any cause. Peripheral blood samples were obtained within one week prior to NAC initiation and again 3 days before surgery. pCR was defined, according to the postoperative pathology, as the breast and lymph nodes free of invasive cancer but allowing carcinoma in situ of the breast (ypT0/Tis, ypN0) [28]. OS was the time from operation to death from any cause or the date of the last follow-up visit. DFS was defined as the time from the date of surgery to the date of local recurrence or distant metastases, death from any cause, or the last follow-up.

### Statistical analysis

All analyses were conducted using SPSS (version 21.0) and R software (version 3.6.1). The thresholds of NLR, LMR and Alb were determined by the maximally selected rank statistics through the maxstat.text function based on the “maxstat” package in R software [29]. Percentages or ranges were applied to describe the different variables. The chi-squared test or Fisher's exact test was applied to assess the differences. The multicollinearity among the different variables was tested by multiple linear regression analysis via the variance inflation factor (VIF), and a VIF ≤ 2 was considered noncollinear [30]. The K-M curves were applied to estimate the survival curves, and the log-rank test was performed to compare them. The proportional hazards (PH) assumption was tested by the log-minus-log-survival (LML) function. The Cox proportional hazards model was used to test the relationships between the variables and OS and DFS. A test was performed to study the interaction between the traditional biomarkers, pCR and mSIS. A nomogram was established on the basis of the multivariate Cox analysis. The concordance index (C-index) was utilized to determine the model’s accuracy, and bootstrapping techniques were used to internally validate the prognostic models. A graphic analysis was performed on the differences between the actual and predicted probabilities obtained from the nomograms. Additionally, prognostic models of the nomogram and clinical TNM stage were investigated by decision curve analysis (DCA). We evaluated the clinical applicability of the nomogram, clinical TNM stage, NLR and Alb by comparing their AUC and net benefits. *P* < 0.05 was considered statistically significant.

## Results

### Baseline characteristics of all malignant breast tumor patients

The optimal thresholds of NLR, LMR and Alb were 2.24, 2.96 and 46.3, respectively, for the outcome OS. The patients were divided into low and high NLR/LMR/Alb value groups for the following analysis. According to the cutoff value, all patients were separated into two groups by SIS (patients with LMR > 2.96 and Alb > 46.3 scored 0; LMR > 2.96 or Alb > 46.3 scored 1; LMR ≤ 2.96 and Alb ≤ 46.3 scored 2; those who scored 0 were placed in the low-risk group, while those scored 1 or 2 were placed in the high-risk group) and mSIS (patients with NLR > 2.24 and Alb ≤ 46.3 scored 0; NLR > 2.24 or Alb ≤ 46.3 scored 1; NLR ≤ 2.24 and Alb > 46.3 scored 2; those who scored 0 were placed in the high-risk group, while those who scored 1 or 2 were placed in the low-risk group).

The median age was 49 years old. A total of 208 (68.2%) patients were in the clinical T2 stage, and 184 (60.3%) patients were in the N2 stage. Fifty-six (18.4%) patients achieved a pCR. A total of 174 (57.0%) patients had the luminal subtype, with a pCR rate of 9.8%; 71 (23.3%) patients had the HER-2 overexpression subtype, with a pCR rate of 25.4%; and 60 (19.7%) patients had the TNBC subtype, with a pCR rate of 35.0%. Only 19 (6.2%) patients received trastuzumab, and 10 of them achieved a pCR. A total of 286 (93.8%) patients did not receive trastuzumab, among whom 46 patients achieved a pCR. A total of 50.6% (88/174) patients with the luminal subtype received adjuvant endocrine therapy, and 49.4% (86/174) patients refused it or failed to comply. A total of 18.3% (11/60) of patients with the TNBC subtype received adjuvant capecitabine, and 81.7% (49/60) of patients refused it or failed to comply.

No relationship was found between mSIS and the clinicopathologic characteristics (*P* > 0.05, Table 1), but mSIS was significantly associated with the LMR (binary), lymphocyte (binary), neutrophil (binary), monocyte (binary), NLR (continuous), LMR (continuous), lymphocyte (continuous), neutrophil (continuous), monocyte (continuous), and albumin (continuous) levels preneoadjuvant chemotherapy, as well as the lymphocyte and neutrophil (binary) levels post neoadjuvant chemotherapy (*P* < 0.05, Table 2). No significant difference was observed between SIS and the clinicopathologic characteristics (*P* > 0.05, Table S1), but SIS was significantly associated with the hemoglobin and albumin (continuous) levels preneoadjuvant chemotherapy and with the albumin levels post neoadjuvant chemotherapy (*P* < 0.05, Table S2).

**Table 1:** Clinicopathologic characteristics of all patients stratified by mSIS

Parameters		High risk	Low risk	*P*
	*N* = 305 (%)	*n* = 65 (%)	*n* = 240 (%)	
Age (median [IQR])	49 [42, 57]	50 [43, 58]	49 [41, 56]	0.053
Age				0.286
≤ 49	161 (52.8)	30 (46.2)	131 (54.6)	
> 49	144 (47.2)	35 (53.8)	109 (45.4)	
Position				0.998
left	176 (57.7)	37 (56.9)	139 (57.9)	
right	129 (42.3)	28 (43.1)	101 (42.1)	
BMI				1
< 24	161 (52.8)	34 (52.3)	127 (52.9)	
≥ 24	144 (47.2)	31 (47.7)	113 (47.1)	
Height				0.168
≤ 1.6	167 (54.8)	41 (63.1)	126 (52.5)	
> 1.6	138 (45.2)	24 (36.9)	114 (47.5)	
Weight				0.479
≤ 61.5	155 (50.8)	30 (46.2)	125 (52.1)	
> 61.5	150 (49.2)	35 (53.8)	115 (47.9)	
Menopause				0.714
no	168 (55.1)	34 (52.3)	134 (55.8)	
yes	137 (44.9)	31 (47.7)	106 (44.2)	
Clinical T stage				0.093
cT1	37 (12.1)	8 (12.3)	29 (12.1)	
cT2	208 (68.2)	39 (60.0)	169 (70.4)	
cT3	55 (18.0)	15 (23.1)	40 (16.7)	
cT4	5 (1.6)	3 (4.6)	2 (0.8)	
Clinical N stage				0.117
cN0	13 (4.3)	3 (4.6)	10 (4.2)	
cN1	29 (9.5)	3 (4.6)	26 (10.8)	
cN2	184 (60.3)	47 (72.3)	137 (57.1)	
cN3	79 (25.9)	12 (18.5)	67 (27.9)	
Clinical TNM stage				0.271
I + II	38 (12.5)	5 (7.7)	33 (13.8)	
III	267 (87.5)	60 (92.3)	207 (86.2)	
Molecular subtype				0.631
luminal A	39 (12.8)	9 (13.8)	30 (12.5)	
luminal B	135 (44.2)	25 (38.5)	110 (45.8)	
HER-2 OE	71 (23.3)	15 (23.1)	56 (23.3)	
TNBC	60 (19.7)	16 (24.6)	44 (18.3)	
ER status				0.479
negative	136 (44.6)	32 (49.2)	104 (43.3)	
positive	169 (55.4)	33 (50.8)	136 (56.7)	
PR status				0.892
negative	169 (55.4)	37 (56.9)	132 (55.0)	
positive	136 (44.6)	28 (43.1)	108 (45.0)	
HER-2 status				0.527
negative	111 (36.4)	23 (35.4)	88 (36.7)	
low expression	87 (28.5)	22 (33.8)	65 (27.1)	
positive	107 (35.1)	20 (30.8)	87 (36.2)	
Ki-67 index				0.188
≤ 14%	103 (33.8)	17 (26.2)	86 (35.8)	
> 14%	202 (66.2)	48 (73.8)	154 (64.2)	
P53 status				0.209
negative	214 (70.2)	41 (63.1)	173 (72.1)	
positive	91 (29.8)	24 (36.9)	67 (27.9)	
Cycle				0.992
< 4	40 (13.1)	8 (12.3)	32 (13.3)	
≥ 4	265 (86.9)	57 (87.7)	208 (86.7)	
pCR				0.379
no	249 (81.6)	56 (86.2)	193 (80.4)	
yes	56 (18.4)	9 (13.8)	47 (19.6)	

*mSIS* Modified systemic inflammation score, *BMI* Body mass index, *HER2-OE* Human epidermal growth factor receptor2 overexpression, *TNBC* Triple negative breast cancer, *ER* Estrogen receptor, *PR* Progesterone receptor, *pCR* Pathologic complete response

**Table 2:** Hematological characteristics of all patients stratified by mSIS

Parameters		High risk	Low risk	*P*
	*N* = 305 (%)	*n* = 65 (%)	*n* = 240 (%)	
**Preneoadjuvant Chemotherapy**				
NLR^a^	-	-	-	-
LMR				< 0.001
≤ 2.96	33 (10.8)	18 (27.7)	15 (6.2)	
> 2.96	272 (89.2)	47 (72.3)	225 (93.8)	
Lymphocyte				< 0.001
≤ 1.96	154 (50.5)	52 (80.0)	102 (42.5)	
> 1.96	151 (49.5)	13 (20.0)	138 (57.5)	
Neutrophil				< 0.001
≤ 3.76	152 (49.8)	11 (16.9)	141 (58.8)	
> 3.76	153 (50.2)	54 (83.1)	99 (41.2)	
Monocyte				0.017
≤ 0.41	155 (50.8)	24 (36.9)	131 (54.6)	
> 0.41	150 (49.2)	41 (63.1)	109 (45.4)	
Hemoglobin				0.070
≤ 135.4	155 (50.8)	40 (61.5)	115 (47.9)	
> 135.4	150 (49.2)	25 (38.5)	125 (52.1)	
Platelet				0.353
≤ 242	154 (50.5)	29 (44.6)	125 (52.1)	
> 242	151 (49.5)	36 (55.4)	115 (47.9)	
Albumin^a^	-	-	-	-
Globulin				0.991
≤ 30	157 (51.5)	34 (52.3)	123 (51.2)	
> 30	148 (48.5)	31 (47.7)	117 (48.8)	
NLR (median [IQR])	1.82 [1.45, 2.46]	2.97 [2.54, 3.51]	1.66 [1.37, 1.98]	< 0.001
LMR (median [IQR])	4.95 [3.75, 6.18]	3.36 [2.90, 4.47]	5.22 [4.26, 6.68]	< 0.001
Lymphocyte (median [IQR])	1.96 [1.61, 2.46]	1.50 [1.31, 1.87]	2.14 [1.77, 2.63]	< 0.001
Neutrophil (median [IQR])	3.77 [2.97, 4.72]	4.83 [4.04, 5.76]	3.55 [2.86, 4.25]	< 0.001
Monocyte (median [IQR])	0.41 [0.34, 0.52]	0.46 [0.38, 0.53]	0.40 [0.33, 0.52]	0.030
Hemoglobin (median [IQR])	135 [129, 142]	134 [126, 141]	136 [130, 142]	0.158
Platelet (median [IQR])	242 [211, 283]	251 [206, 299]	239 [211, 279]	0.523
Albumin (median [IQR])	45.0 [43.0, 46.6]	44.0 [42.2, 45.0]	45.0 [43.0, 47.0]	< 0.001
Globulin (median [IQR])	30 [28, 33]	30 [28.9, 33.0]	30 [27.8, 33.0]	0.518
**Post Neoadjuvant Chemotherapy**				
Lymphocyte				0.003
≤ 1.54	154 (50.5)	44 (67.7)	110 (45.8)	
> 1.54	151 (49.5)	21 (32.3)	130 (54.2)	
Neutrophil				0.023
≤ 3.75	153 (50.2)	24 (36.9)	129 (53.8)	
> 3.75	152 (49.8)	41 (63.1)	111 (46.2)	
Monocyte				0.504
≤ 0.53	159 (52.1)	31 (47.7)	128 (53.3)	
> 0.53	146 (47.9)	34 (52.3)	112 (46.7)	
Hemoglobin				0.757
≤ 117	153 (50.2)	31 (47.7)	122 (50.8)	
> 117	152 (49.8)	34 (52.3)	118 (49.2)	
Platelet				0.251
≤ 271	153 (50.2)	28 (43.1)	125 (52.1)	
> 271	152 (49.8)	37 (56.9)	115 (47.9)	
Albumin				0.983
≤ 46.3	256 (83.9)	54 (83.1)	202 (84.2)	
> 46.3	49 (16.1)	11 (16.9)	38 (15.8)	
Globulin				0.609
≤ 28	158 (51.8)	36 (55.4)	122 (50.8)	
> 28	147 (48.2)	29 (44.6)	118 (49.2)	
Lymphocyte (median [IQR])	1.54 [1.19, 1.87]	1.24 [1.07, 1.61]	1.60 [1.31, 1.95]	< 0.001
Neutrophil (median [IQR])	3.75 [2.81, 4.81]	3.99 [2.95, 5.06]	3.58 [2.78, 4.65]	0.124
Monocyte (median [IQR])	0.53 [0.42, 0.68]	0.55 [0.45, 0.69]	0.52 [0.42, 0.68]	0.625
Hemoglobin (median [IQR])	117 [110, 124]	118 [109, 123]	117 [110, 124]	0.912
Platelet (median [IQR])	271 [230, 335]	288 [235, 338]	269 [229, 332]	0.402
Albumin (median [IQR])	43.0 [41.0, 45.0]	43.0 [41.0, 45.0]	43.0 [41.0, 45.0]	0.579
Globulin (median [IQR])	28.0 [25.0, 31.0]	28.0 [25.0, 30.6]	28.0 [25.1, 31.0]	0.620

*mSIS* Modified systemic inflammation score, *NLR* Neutrophil to lymphocyte ratio, *LMR* Lymphocyte to monocyte ratio

^a^preneoadjuvant chemotherapy NLR and Alb were excluded from the correlation analysis because they are part of the mSIS

### Cox regression survival analysis

The PH assumption was tested before the survival analysis. The LML function demonstrated that the log-minus-log curves of the low and high mSIS groups were almost parallel (Figure S1).

The univariate analysis for OS showed that OS was significantly associated with age, menopause, mSIS, NLR, LMR, hemoglobin, clinical T stage and pCR (*P* < 0.05). Then, the multicollinearity among these parameters was tested. The variables, including age, menopause and NLR, were excluded from the multivariate analysis because their VIF value was > 2, and other variables were incorporated. The results demonstrated that only mSIS, clinical T stage, and pCR were independently correlated with OS (HR = 0.69, 95% CI: 0.38–0.96, *P* = 0.0322; HR = 3.43, 95% CI: 1.05–11.2, *P* = 0.0410; HR = 0.40, 95% CI: 0.20–0.80, *P* = 0.0096, respectively, Table 3). In the univariate and multivariate analyses for DFS, only LMR and pCR were independently correlated with DFS (HR = 0.57, 95% CI: 0.36–0.89, *P* = 0.0142; HR = 0.43, 95% CI: 0.26–0.72, *P* = 0.0013, respectively, Table 4). No significant association was observed between SIS, OS and DFS (*P* > 0.05, Tables 3 and 4).

**Table 3:** Univariate and multivariate analysis for OS

Parameters	Univariate analysis		Multivariate analysis	
	HR (95% CI)	*P*	HR (95% CI)	*P*
Age (≤ 49 vs. > 49) ^a^	1.63 (1.09–2.45)	**0.019**		
Position (left vs. right)	1.05 (0.7–1.57)	0.830		
BMI (< 24 vs. ≥ 24)	1.26 (0.84–1.88)	0.269		
Height (≤ 1.6 vs. > 1.6)	0.8 (0.53–1.2)	0.277		
Weight (≤ 61.5 vs. > 61.5)	1.13 (0.76–1.69)	0.551		
Menopause (no vs. yes) ^a^	1.74 (1.16–2.6)	**0.008**		
**Preneoadjuvant chemotherapy hematological parameters**				
mSIS (high risk vs. low risk)	0.53 (0.34–0.82)	**0.004**	0.60 (0.38–0.96)	**0.0322**
SIS (high risk vs. low risk)	1.17 (0.74–1.85)	0.513		
NLR (≤ 2.24 vs. > 2.24) ^a^	1.88 (1.25–2.83)	**0.002**		
LMR (≤ 2.96 vs. > 2.96)	0.55 (0.32–0.94)	**0.028**	0.70 (0.39–1.24)	0.2216
Lymphocyte (≤ 1.96 vs. > 1.96)	0.88 (0.59–1.32)	0.532		
Neutrophil (≤ 3.76 vs. > 3.76)	1.12 (0.75–1.67)	0.591		
Monocyte (≤ 0.41 vs. > 0.41)	0.85 (0.57–1.28)	0.437		
Hemoglobin (≤ 135.4 vs. > 135.4)	0.65 (0.43–0.98)	**0.041**	0.76 (0.50–1.16)	0.2085
Platelet (≤ 242 vs. > 242)	0.99 (0.66–1.47)	0.944		
Albumin (≤ 46.3 vs. > 46.3)	1.27 (0.82–1.97)	0.279		
Globulin (≤ 30 vs. > 30)	1.03 (0.69–1.53)	0.900		
Clinical T stage (1 vs. 2)	1.1 (0.56–2.15)	0.776	0.90 (0.46–1.78)	0.7663
Clinical T stage (1 vs. 3)	1.38 (0.64–2.96)	0.414	1.05 (0.48–2.28)	0.9050
Clinical T stage (1 vs. 4)	4.51 (1.41–14.41)	**0.011**	3.43 (1.05–11.2)	**0.0410**
Clinical N stage (0 vs. 1)	2.08 (0.59–7.39)	0.255		
Clinical N stage (0 vs. 2)	1.37 (0.43–4.38)	0.595		
Clinical N stage (0 vs. 3)	1.72 (0.52–5.68)	0.375		
Clinical TNM stage (I + II vs. III)	0.82 (0.46–1.44)	0.486		
Subtype (luminal A vs. B)	1.84 (0.87–3.89)	0.110		
Subtype (luminal A vs. HER-2 OE)	1.15 (0.49–2.66)	0.752		
Subtype (luminal A vs. TNBC)	1.91 (0.85–4.32)	0.119		
ER (negative vs. positive)	1.16 (0.77–1.74)	0.482		
PR (negative vs. positive)	1.05 (0.7–1.57)	0.812		
HER-2 (negative vs. low expression)	0.94 (0.58–1.53)	0.798		
HER-2 (negative vs. positive)	0.74 (0.46–1.21)	0.232		
Ki-67 (≤ 14% vs. > 14%)	1.23 (0.79–1.89)	0.358		
P53 (negative vs. positive)	1.17 (0.76–1.81)	0.466		
**Post neoadjuvant chemotherapy hematological parameters**				
Lymphocyte (≤ 1.54 vs. > 1.54)	1.02 (0.68–1.53)	0.914		
Neutrophil (≤ 3.75 vs. > 3.75)	1.06 (0.71–1.58)	0.793		
Monocyte (≤ 0.53 vs. > 0.53)	0.79 (0.53–1.19)	0.255		
Hemoglobin (≤ 117 vs. > 117)	0.75 (0.5–1.13)	0.172		
Platelet (≤ 271 vs. > 271)	1.24 (0.83–1.86)	0.296		
Albumin (≤ 46.3 vs. > 46.3)	1.03 (0.6–1.76)	0.922		
Globulin (≤ 28 vs. > 28)	0.92 (0.61–1.38)	0.688		
Cycle (< 4 vs. ≥ 4)	0.74 (0.43–1.26)	0.264		
pCR (no vs. yes)	0.41 (0.21–0.82)	**0.012**	0.40 (0.20–0.80)	**0.0096**

^a^Age, menopause and NLR were excluded from the multivariate analysis because they had a VIF value > 2

**Table 4:** Univariate and multivariate analysis for DFS

Parameters	Univariate analysis		Multivariate analysis	
	HR (95% CI)	*P*	HR (95% CI)	*P*
Age (≤ 49 vs. > 49)	1.08 (0.79–1.48)	0.638		
Position (left vs. right)	0.92 (0.66–1.27)	0.602		
BMI (< 24 vs. ≥ 24)	1.3 (0.94–1.79)	0.109		
Height (≤ 1.6 vs. > 1.6)	0.93 (0.67–1.28)	0.648		
Weight (≤ 61.5 vs. > 61.5)	1.29 (0.93–1.77)	0.123		
Menopause (no vs. yes)	1.22 (0.89–1.68)	0.218		
**Preneoadjuvant chemotherapy hematological parameters**				
mSIS (high risk vs. low risk)	0.72 (0.50–1.04)	0.078		
SIS (high risk vs. low risk)	1.10 (0.75–1.60)	0.629		
NLR (≤ 2.24 vs. > 2.24)	1.39 (1–1.94)	0.052		
LMR (≤ 2.96 vs. > 2.96)	0.6 (0.38–0.95)	**0.028**	0.57 (0.36–0.89)	**0.0142**
Lymphocyte (≤ 1.96 vs. > 1.96)	0.97 (0.7–1.33)	0.834		
Neutrophil (≤ 3.76 vs. > 3.76)	1 (0.73–1.37)	0.992		
Monocyte (≤ 0.41 vs. > 0.41)	0.91 (0.66–1.25)	0.557		
Hemoglobin (≤ 135.4 vs. > 135.4)	0.66 (0.48–0.91)	**0.012**	0.78 (0.55–1.10)	0.1550
Platelet (≤ 242 vs. > 242)	0.98 (0.71–1.35)	0.907		
Albumin (≤ 46.3 vs. > 46.3)	1.2 (0.84–1.7)	0.32		
Globulin (≤ 30 vs. > 30)	1 (0.73–1.38)	1		
Clinical T stage (1 vs. 2)	0.82 (0.51–1.33)	0.417		
Clinical T stage (1 vs. 3)	1.11 (0.64–1.95)	0.707		
Clinical T stage (1 vs. 4)	2.39 (0.81–7)	0.113		
Clinical N stage (0 vs. 1)	2.48 (0.83–7.38)	0.102		
Clinical N stage (0 vs. 2)	1.75 (0.64–4.78)	0.273		
Clinical N stage (0 vs. 3)	2.5 (0.9–6.96)	0.08		
Clinical TNM stage (I + II vs. III)	1 (0.62–1.62)	0.994		
Subtype (luminal A vs. B)	1.23 (0.73–2.06)	0.438		
Subtype (luminal A vs. HER-2 OE)	1.04 (0.58–1.84)	0.904		
Subtype (luminal A vs. TNBC)	1.1 (0.61–1.98)	0.761		
ER (negative vs. positive)	1.05 (0.76–1.45)	0.751		
PR (negative vs. positive)	1 (0.73–1.38)	0.981		
HER-2 (negative vs. low expression)	0.95 (0.64–1.4)	0.788		
HER-2 (negative vs. positive)	0.85 (0.58–1.24)	0.396		
Ki-67 (≤ 14% vs. > 14%)	0.98 (0.7–1.37)	0.912		
P53 (negative vs. positive)	0.86 (0.6–1.23)	0.409		
**Post neoadjuvant chemotherapy hematological parameters**				
Lymphocyte (≤ 1.54 vs. > 1.54)	0.94 (0.68–1.29)	0.699		
Neutrophil (≤ 3.75 vs. > 3.75)	1.11 (0.81–1.53)	0.519		
Monocyte (≤ 0.53 vs. > 0.53)	0.82 (0.6–1.14)	0.238		
Hemoglobin (≤ 117 vs. > 117)	0.69 (0.5–0.95)	**0.025**	0.75 (0.53–1.06)	0.0982
Platelet (≤ 271 vs. > 271)	1.07 (0.78–1.47)	0.672		
Albumin (≤ 46.3 vs. > 46.3)	0.96 (0.62–1.49)	0.855		
Globulin (≤ 28 vs. > 28)	0.82 (0.6–1.14)	0.239		
Cycle (< 4 vs. ≥ 4)	0.93 (0.59–1.46)	0.746		
pCR (no vs. yes)	0.44 (0.27–0.73)	**0.001**	0.43 (0.26–0.72)	**0.0013**

**Fig. 1:**
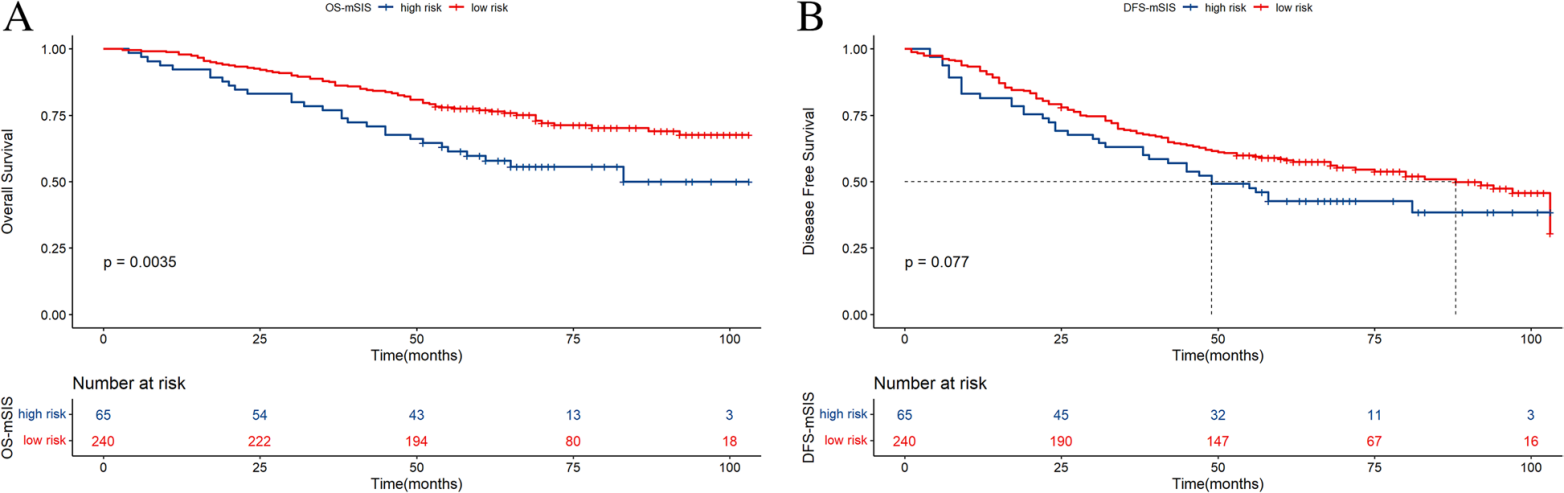
Kaplan‒Meier survival curves of breast cancer patients who underwent neoadjuvant chemotherapy for OS (**A**) and DFS (**B**) stratified by mSIS

For mSIS, the patients in the low-risk group had better 5- and 8-year OS rates than those in the high-risk group (59.8% vs. 77.0%; 50.1% vs. 67.7%; X^2^ = 8.5, *P* = 0.0035, respectively, Fig. 1A). A similar trend was observed for the 5- and 8-year DFS rates (42.8% vs. 58.5%; 38.5 vs. 47.4%; X^2^ = 3.1, *P* = 0.077, respectively, Fig. 1B).

### The interactions between mSIS, traditional biomarkers and pCR

Traditional biomarkers (ER, PR, HER-2, Ki-67) and pCR are associated with the prognosis of malignant breast tumor patients. To identify their association with the mSIS, statistical tests for interactions were performed. In the traditional biomarker subgroups, no significant difference was found for OS and DFS in the different mSIS groups (*P* value for interaction > 0.05).

In the pCR subgroup, patients in the mSIS low-risk group had a worse OS (HR = 1.558, 95% CI: 0.195–12.47, *P* value for interaction = 0.033; Table 5), but those in the mSIS low-risk group had a better DFS (HR = 0.987, 95% CI: 0.275–3.540, *P* value for interaction = 0.033; Table 6).

**Table 5:** Stratified association between mSIS and OS by traditional biomarkers and pCR

Parameters	mSIS high risk	mSIS low risk	*P* value	*P* value for interaction
	HR (95% CI)			
ER				0.320
negative	1 (reference)	0.628 (0.318–1.240)	0.180	
positive	1 (reference)	0.440 (0.248–0.780)	0.005	
PR				0.161
negative	1 (reference)	0.691 (0.373–1.278)	0.239	
positive	1 (reference)	0.377 (0.202–0.706)	0.002	
HER-2				0.181
negative	1 (reference)	0.397 (0.203–0.777)	0.007	
low expression	1 (reference)	0.381 (0.180–0.807)	0.012	
positive	1 (reference)	1.372 (0.477–3.949)	0.558	
Ki-67				0.278
≤ 14%	1 (reference)	0.621 (0.266–1.450)	0.271	
> 14%	1 (reference)	0.510 (0.305–0.852)	0.010	
pCR				**0.033**
no	1 (reference)	0.491 (0.313–0.773)	0.002	
yes	1 (reference)	1.558 (0.195–12.47)	0.676

**Table 6:** Stratified association between mSIS and DFS by traditional biomarkers and pCR

Parameters	mSIS high risk	mSIS low risk	*P* value	*P* value for interaction
	HR (95% CI)			
ER				0.622
negative	1 (reference)	0.741 (0.429–1.279)	0.282	
positive	1 (reference)	0.699 (0.423–1.152)	0.160	
PR				0.530
negative	1 (reference)	0.738 (0.449–1.215)	0.232	
positive	1 (reference)	0.699 (0.404–1.207)	0.199	
HER-2				0.412
negative	1 (reference)	0.519 (0.295–0.914)	0.023	
low expression	1 (reference)	0.729 (0.381–1.394)	0.339	
positive	1 (reference)	1.078 (0.504–2.304)	0.847	
Ki-67				0.180
≤ 14%	1 (reference)	0.879 (0.442–1.749)	0.714	
> 14%	1 (reference)	0.641 (0.413–0.996)	0.048	
pCR				**0.004**
no	1 (reference)	0.722 (0.491–1.061)	0.097	
yes	1 (reference)	0.987 (0.275–3.540)	0.984	

### The relationship between OS, DFS and mSIS in malignant breast tumor patients with different pCR statuses

**Fig. 2:**
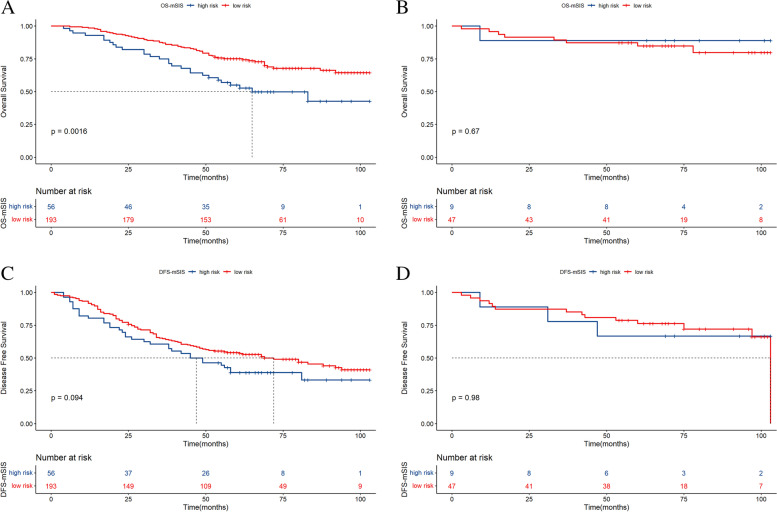
Kaplan‒Meier survival curves of breast cancer patients with nonpCR for OS (**A**) and DFS (**B**) stratified by mSIS. The survival curves of breast cancer patients with pCR for OS (**C**) and DFS (**D**) stratified by mSIS

Tables 3 and 4 demonstrate that pCR was independently correlated with OS and DFS, so we explored the predictive ability of mSIS in different pCR status groups. The results demonstrated that patients who achieved a pCR had higher 5- and 8-year OS and DFS rates (*P* = 0.0091; *P* = 0.0011, respectively, Figure S2A, B). In the nonpCR subgroup, patients in the mSIS low-risk group had higher 5- and 8-year OS rates than those in the mSIS high-risk group (55.0% vs. 75.1%; 42.8% vs. 64.4%; X^2^ = 9.9, *P* = 0.0016, respectively, Fig. 2A). A similar trend was observed for the 5- and 8-year DFS rates (38.8% vs. 54.2%; 33.3% vs. 41.0%; X^2^ = 2.8, *P* = 0.094, respectively, Fig. 2B). In the pCR subgroup, the different mSIS groups showed no significant difference in OS and DFS (*P* > 0.05, Fig. 2C, D).

### The prognostic value of different combinations of mSIS and pCR

Our results showed that patients who achieved a pCR had better OS and DFS. Thus, we assessed the association of mSIS with the outcome according to the different pCR statuses.

**Fig. 3:**
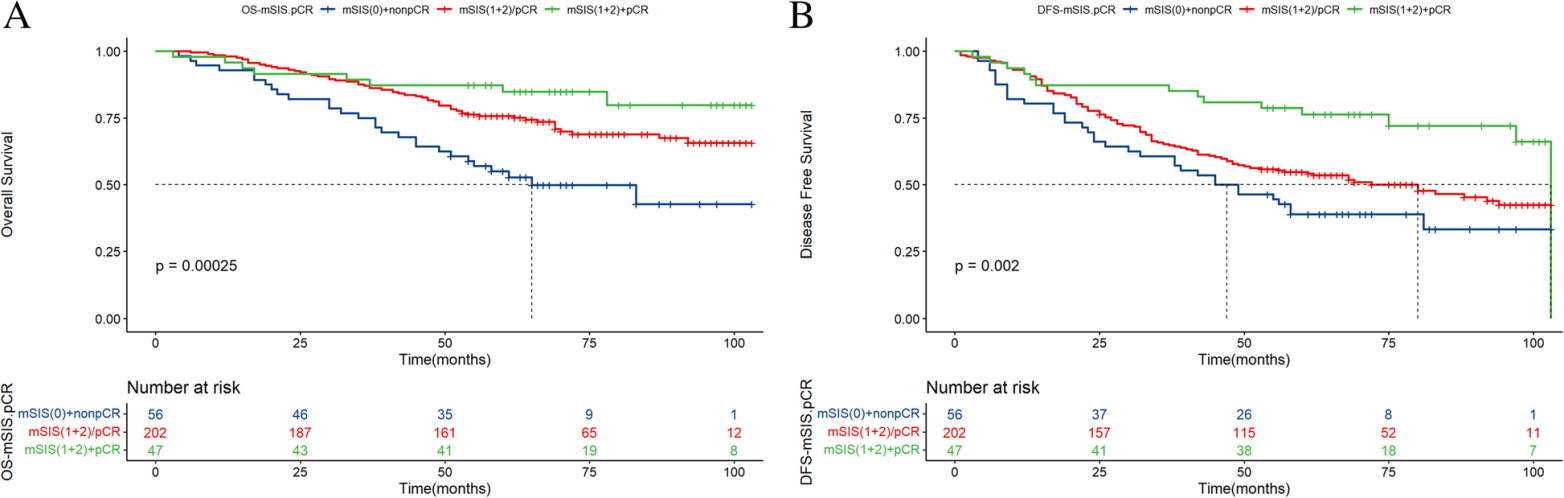
Risk stratification combining mSIS and pCR in relation to OS and DFS of breast cancer patients. Kaplan‒Meier curves of three risk groups for OS (**A**) and DFS (**B**)

The stratification by the combination of mSIS and pCR divided the patients into three subgroups: mSIS (0 score) + nonpCR, mSIS (1 + 2 score) or pCR, and mSIS (1 + 2 score) + pCR. These results demonstrated that patients in the mSIS (1 + 2 score) + pCR subgroup had the highest 5- and 8-year OS and DFS rates (OS: 55.0% vs. 75.7% vs. 84.8, 42.8% vs. 65.7% vs. 79.8%, X^2^ = 16.6, *P* = 0.00025; DFS: 38.8% vs. 54.7% vs. 76.3%, 33.3% vs. 42.3 vs. 72.1%, X^2^ = 12.4, *P* = 0.002; Fig. 3A, B).

### Development and validation of nomograms for OS

**Fig. 4:**
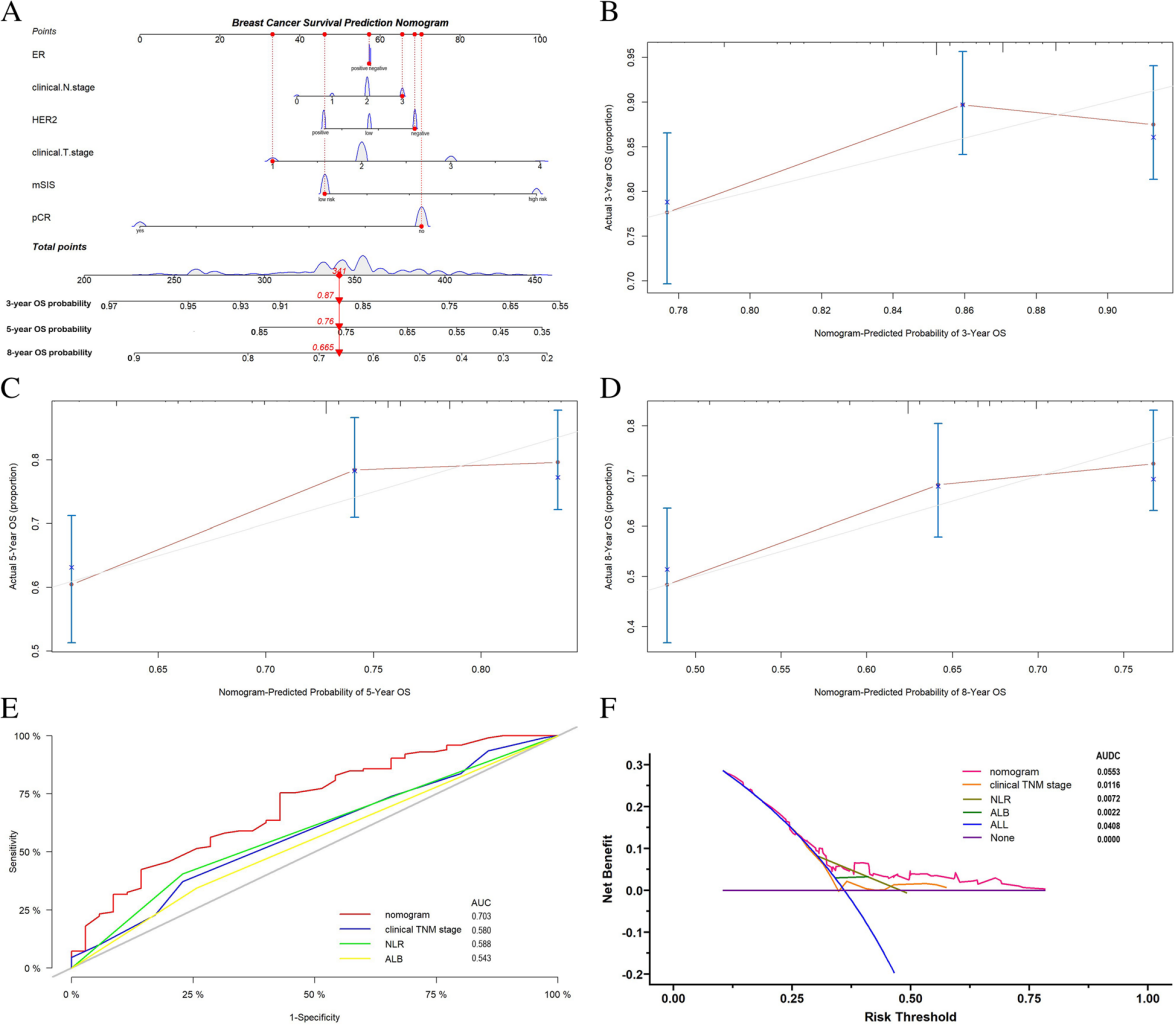
Nomogram to predict the overall survival of breast cancer patients undergoing neoadjuvant chemotherapy. A nomogram was generated based on ER, HER2, clinical T stage, clinical N stage, pCR and mSIS to predict OS (**A**). The 3-, 5-year and 8-year OS rates of the breast cancer patients predicted by the nomogram were highly consistent with the actual observed values (**B**, **C**, **D**). Comparison of the predictive ability between the nomogram, clinical TNM stage, NLR and ALB (**E**). Decision curve analyses (DCA) for the prognostic models of the nomogram, clinical TNM stage, NLR and ALB (**F**). The nomogram maps predict the probabilities onto the points on a scale from 0 to 100 and can be interpreted by adding the points together that correspond to the predicted probability. The total points were converted into the probabilities of survival for breast cancer patients after 3, 5 and 8 years

The variables with *P* < 0.05 in the multivariate analysis for OS were applied to construct a nomogram for individualized OS prediction. ER, HER-2 and nodal status are correlated with OS [31, 32], so the nomogram still included these variables even though our study did not observe a significant difference (Fig. 4A). The C-index was 0.631, and the internal validation results were similar when bootstrapping was utilized (0.585). The 3-, 5-, and 8-year OS predictions were highly consistent with the actual observations (Fig. 4B, C, D). The nomogram had the best predictive ability for OS compared to clinical TNM stage, NLR and Alb (AUC = 0.703; AUC = 0.580; AUC = 0.588; AUC = 0.543, respectively, Fig. 4E). According to the decision curve analyses (DCA) for the models using clinical TNM stage, NLR and ALB, all four models showed a positive net benefit in predicting OS, and among them, the nomogram had the best clinical applicability because of a larger area under the decision curve (AUDC) for OS (AUDC = 0.0553, AUDC = 0.0116, AUDC = 0.0072, and AUDC = 0.0022, respectively, Fig. 4F).

## Discussion

This research demonstrated that the mSIS is significantly associated with OS and is an independent predictor of OS. Patients in the mSIS low-risk group had a longer OS. The mSIS-based nomogram outperformed the clinical TNM stage in predicting the individualized OS of patients undergoing NAC. In the future, more rigorous follow-ups and more information about patients receiving adjuvant therapy are required. The nomogram needs to be validated and adjusted with data from a large multicenter sample, which will enable a more accurate prediction of individual survival.

In recent decades, systemic inflammation has drawn wide attention from scientists, and cancer-related inflammatory responses are acknowledged as a factor promoting malignant tumor progression [33, 34]. The tumor microenvironment is influenced by cancer-related inflammation, and the inflammation occurs prior to the manifestation of malignant changes [35, 36]. In addition, cancer-related inflammation could lead to changes in the peripheral blood cell counts, such as lymphocytes and monocytes [37]. Because these inflammatory and immunity factors can promote or inhibit the development of tumors, the survival outcome of patients can also be affected. Infiltrating neutrophils suppress inflammatory factors in the tumor microenvironment, which may allow tumor cells to evade detection by immune cells [38, 39]. Through the release of inflammatory mediators (such as neutrophil elastase, interleukin-8, matrix metalloproteinase-9, and vascular endothelial growth factor), neutrophils can also influence the proliferation and metastasis of tumors [38, 40]. In addition, tumor angiogenesis, inflammation, and metastasis can also be induced by monocytes to inhibit the immune system [41, 42]. There has been increasing evidence that monocytes are essential premetastatic promoters and are rapidly recruited from the bone marrow, mainly via the CCL2/CCR2 axis, to premetastatic niches, where they promote tumor colonization by secreting angiogenic factors such as VEGFA [43–45]. In contrast, as essential immune cells, lymphocytes can mediate cellular immunity suppression or release granzymes to prevent tumor progression [46, 47].

The magnitude of tumor infiltrating lymphocytes (TILs) is variable in different breast cancer subtypes, and it can improve the clinical response in concert with chemotherapy and immune checkpoint inhibitor therapy [48]. Many studies have demonstrated that more TILs are correlated with a better local response to NAC and a better prognosis of breast cancer patients [49, 50]. Different from these inflammatory parameters, albumin is one of the nutritional indicators, and malnutrition weakens the immunological and phagocytosis mechanisms of the human body, increasing the risk of infection and other diseases [51].

Based on the above inflammatory and nutritional parameters, a systemic inflammation score system (SIS, including LMR and Alb) and a modified systemic inflammation score system (mSIS, including NLR and Alb) were constructed. Zhang-Zan Huang and his team found that breast cancer patients in the high-SIS group had worse OS [15], but they did not investigate the predictive ability of SIS among malignant breast tumor patients who underwent NAC. mSIS is also a good biomarker for many cancers [16, 17], but it has not been used for malignant breast tumors thus far, particularly for patients who underwent NAC.

Our research explored the predictive ability of these two scoring systems in breast cancer patients who underwent NAC. A significant relationship between SIS and OS and DFS was not observed because most patients who underwent NAC were suffering from locally advanced or advanced breast cancer, while those who underwent surgery directly had an early TNM stage. Moreover, the progression of cancer could induce inflammation, leading to a change in blood parameters [37]. Therefore, SIS may not be suitable for predicting the outcome of malignant breast cancer among patients who underwent NAC. However, the small sample size may explain the negative result for SIS.

We found that mSIS was an independent predictor of the outcome of malignant breast cancer patients who underwent NAC. Patients in the low mSIS risk group had better 5- and 8-year OS rates than those in the high-risk group, and a similar trend was also observed for 5- and 8-year DFS rates.

In recent years, several gene detection methods, such as Oncotype DX and MammaPrint Assay, have been applied to identify patients with different prognoses to guide choices about their chemotherapy [52, 53]. As these methods are applied based on pathological examination results, they could be considered a combination of genetics and histology. To date, no studies have combined an inflammatory parameter with traditional biomarkers, including pCR, so we conducted an exploratory analysis to assess the interactions between mSIS, traditional biomarkers and pCR. Surprisingly, in the pCR subgroup, patients in the mSIS low-risk group who achieved a pCR had a worse OS (HR = 1.558, 95% CI: 0.195–12.47, *P* value for interaction = 0.033; Table 5). Based on these results, we combined the mSIS with pCR, and then all patients were divided into three groups based on their different mSIS and pCR statuses. These results demonstrated that patients in the mSIS (1 + 2 score) + pCR subgroup had the highest 5- and 8-year OS and DFS rates (*P* = 0.002). Therefore, a combination of mSIS and pCR could be a good choice to predict the outcome of breast cancer patients.

Nomograms have been applied to predict individualized survival in various cancers [54, 55]. In this research, a nomogram was also established based on three independent predictors, i.e., mSIS, clinical T stage and pCR. Through internal validation and graph calibration, these nomograms demonstrate good reliability. In addition, compared to the clinical TNM stage, NLR and Alb, this nomogram demonstrated a better predictive ability and clinical applicability. Thus, a nomogram based on mSIS is a good model to predict individualized survival of malignant breast cancer patients who underwent NAC.

To the best of our knowledge, this study is the first to explore the link between SIS, mSIS and breast cancer patients undergoing NAC. There are, however, several limitations. First, this is a single-institute study, so multi-institute samples are needed. Second, the nomogram needs to undergo external validation in the future as it has only been subjected to internal validation. Third, with an increasing number of new drugs available and reductions in the price of the drugs (such as trastuzumab), patients have been given more choices for their treatment, and their outcomes have improved significantly. Therefore, exploring the prognostic value of the mSIS in a sample containing patients who received trastuzumab is necessary. Fourth, some patients refused adjuvant therapy after undergoing NAC and surgery. Therefore, updated and more complete data are needed to further identify the reliability of the mSIS.

## Conclusions

The mSIS is a useful prognostic tool for malignant breast tumor patients who underwent NAC, but the SIS is not. A combination of the mSIS and pCR is helpful for improving the accuracy of predicting a pCR. The nomogram developed in this study based on mSIS is recommended for predicting the individualized OS of breast cancer patients who underwent NAC. As a convenient and inexpensive indicator, it should be promoted and applied in clinical work.

## Supplementary Information

**Additional file 1: Figure S1.** The log-minus-log curves of low and high mSIS groups. **Figure S2.** Kaplan-Meier survival curves of breast cancer patients underwent neoadjuvant chemo-therapy with different pCR status for OS (A) and DFS (B). **Table S1.** Clinicopathologic characteristics of all patients divided by SIS. **Table S2.** Hematological characteristics of all patients divided by SIS.

## Data Availability

The datasets used and/or analyzed during the current study are not publicly available due to the privacy policy of our hospital but are available from the corresponding author upon reasonable request.
