# Supplementary material for: Prognostic value of a modified systemic inflammation score in breast cancer patients who underwent neoadjuvant chemotherapy
Source: BMC Cancer. 2022 Dec 2;22:1249. doi: 10.1186/s12885-022-10291-2 (PMC9717545; doi:10.1186/s12885-022-10291-2)
Supplement: Supplementary file 1 — Additional file 1: Figure S1. The log-minus-log curves of low and high mSIS groups. Figure S2. Kaplan-Meier survival curves of breast cancer patients underwent neoadjuvant chemo-therapy with different pCR status for OS (A) and DFS (B). Table S1. Clinicopathologic characteristics of all patients divided by SIS. Table S2. Hematological characteristics of all patients divided by SIS. [file 12885_2022_10291_MOESM1_ESM.pdf]

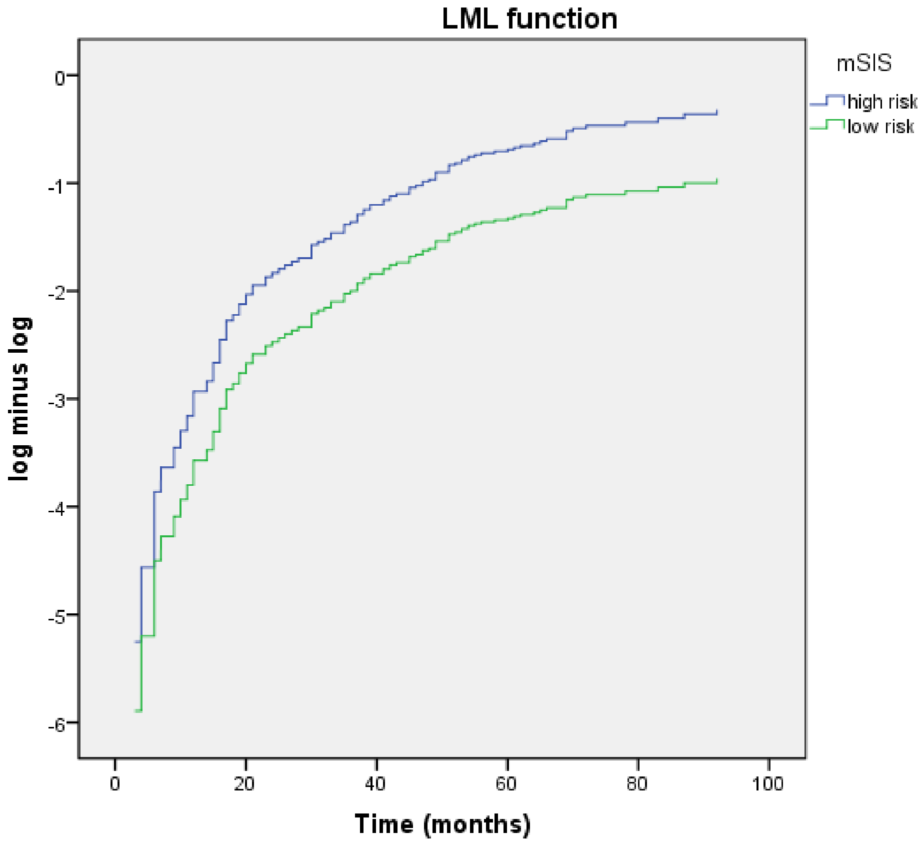

**Figure Supplement 1** The log-minus-log curves of low and high mSIS groups

A

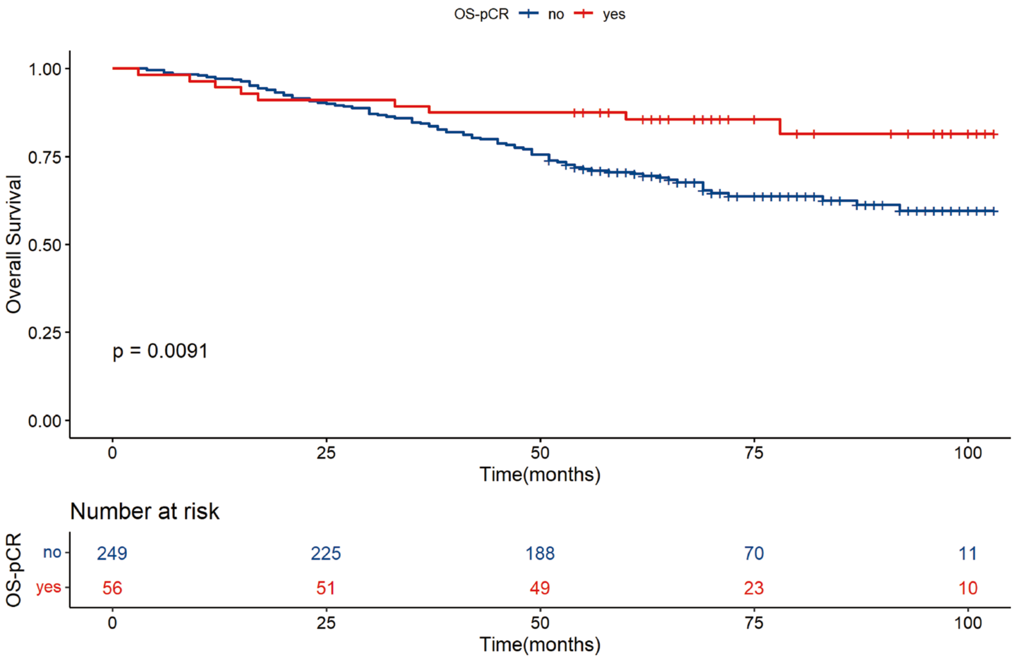


B

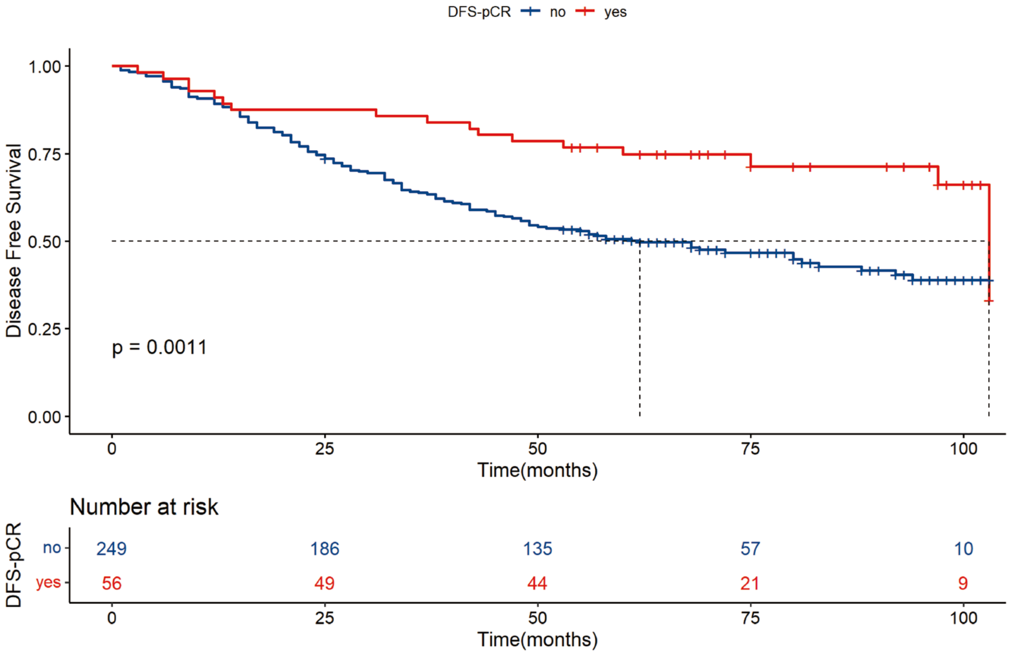


**Figure S2** Kaplan-Meier survival curves of breast cancer patients underwent neoadjuvant chemotherapy with different pCR status for OS (A) and DFS (B).

**Table S1 Clinicopathologic characteristics of all patients divided by SIS**

| Parameters | N=305 (%) | High risk n=236 (%) | Low risk n=69 (%) | P |
|---|---|---|---|---|
| Age (median [IQR]) | 49 [42, 57] | 49 [43, 57] | 46 [38, 56] | 0.050 |
| Age | | | | 0.399 |
| ≤49 | 161 (52.8) | 121 (51.3) | 40 (58.0) | |
| ＞49 | 144 (47.2) | 115 (48.7) | 29 (42.0) | |
| Position | | | | 0.358 |
| left | 176 (57.7) | 140 (59.3) | 36 (52.2) | |
| right | 129 (42.3) | 96 (40.7) | 33 (47.8) | |
| BMI | | | | 0.569 |
| ＜24 | 161 (52.8) | 122 (51.7) | 39 (56.5) | |
| ≥24 | 144 (47.2) | 114 (48.3) | 30 (43.5) | |
| Height | | | | 1 |
| ≤1.6 | 167 (54.8) | 129 (54.7) | 38 (55.1) | |
| ＞1.6 | 138 (45.2) | 107 (45.3) | 31 (44.9) | |
| Weight | | | | 0.505 |
| ≤61.5 | 155 (50.8) | 117 (49.6) | 38 (55.1) | |
| ＞61.5 | 150 (49.2) | 119 (50.4) | 31 (44.9) | |
| Menopause | | | | 0.681 |
| no | 168 (55.1) | 128 (54.2) | 40 (58.0) | |
| yes | 137 (44.9) | 108 (45.8) | 29 (42.0) | |
| clinical T stage | | | | 0.433 |
| cT1 | 37 (12.1) | 26 (11.0) | 11 (15.9) | |
| cT2 | 208 (68.2) | 160 (67.8) | 48 (69.6) | |
| cT3 | 55 (18.0) | 45 (19.1) | 10 (14.5) | |
| cT4 | 5 (1.6) | 5 (2.1) | 0 (0.0) | |
| clinical N stage | | | | 0.247 |
| cN0 | 13 (4.3) | 9 (3.8) | 4 (5.8) | |
| cN1 | 29 (9.5) | 19 (8.1) | 10 (14.5) | |
| cN2 | 184 (60.3) | 148 (62.7) | 36 (52.2) | |
| cN3 | 79 (25.9) | 60 (25.4) | 19 (27.5) | |
| clinical TNM stage | | | | 0.106 |
| I+II | 38 (12.5) | 25 (10.6) | 13 (18.8) | |

| | | | | |
|---|---|---|---|---|
| III | 267 (87.5) | 211 (89.4) | 56 (81.2) | |
| Molecular Subtype | | | | 0.197 |
| luminal A | 39 (12.8) | 34 (14.4) | 5 (7.2) | |
| luminal B | 135 (44.3) | 98 (41.5) | 37 (53.6) | |
| HER-2 OE | 71 (23.3) | 58 (24.6) | 13 (18.8) | |
| TNBC | 60 (19.7) | 46 (19.5) | 14 (20.3) | |
| ER status | | | | 0.727 |
| negative | 136 (44.6) | 107 (45.3) | 29 (42.0) | |
| positive | 169 (55.4) | 129 (54.7) | 40 (58.0) | |
| PR status | | | | 0.727 |
| negative | 169 (55.4) | 129 (54.7) | 40 (58.0) | |
| positive | 136 (44.6) | 107 (45.3) | 29 (42.0) | |
| HER-2 status | | | | 0.995 |
| negative | 111 (36.4) | 86 (36.4) | 25 (36.2) | |
| low expression | 87 (28.5) | 67 (28.4) | 20 (29.0) | |
| positive | 107 (35.1) | 83 (35.2) | 24 (34.8) | |
| Ki-67 index | | | | 0.073 |
| ≤14% | 103 (33.8) | 73 (30.9) | 30 (43.5) | |
| ＞14% | 202 (66.2) | 163 (69.1) | 39 (56.5) | |
| P53 status | | | | 0.128 |
| negative | 214 (70.2) | 160 (67.8) | 54 (78.3) | |
| positive | 91 (29.8) | 76 (32.2) | 15 (21.7) | |
| Cycle | | | | 0.301 |
| ＜4 | 40 (13.1) | 34 (14.4) | 6 (8.7) | |
| ≥4 | 265 (86.9) | 202 (85.6) | 63 (91.3) | |
| pCR | | | | 0.769 |
| no | 249 (81.6) | 194 (82.2) | 55 (79.7) | |
| yes | 56 (18.4) | 42 (17.8) | 14 (20.3) | |

Abbreviation: SIS, systemic inflammation score; BMI, body mass index; HER2-OE, human epidermal growth factor receptor2 over expression; TNBC, triple negative breast cancer; ER, estrogen receptor; PR, progesterone receptor; pCR, pathologic complete response.

**Table S2 Hematological characteristics of all patients divided by SIS**

| Parameters | | High risk | Low risk | P |
|---|---|---|---|---|
| | N=305 (%) | n=236 (%) | n=69 (%) | |
| **Pre-Neoadjuvant Chemotherapy** | | | | |
| NLR | | | | 0.82 |
| | 211 (69.2) | 162 (68.6) | 49 (71.0) | |
| | 94 (30.8) | 74 (31.4) | 20 (29.0) | |
| LMR* | - | - | - | - |
| Lymphocyte | | | | 0.856 |
| ≤1.96 | 154 (50.5) | 118 (50.0) | 36 (52.2) | |
| ＞1.96 | 151 (49.5) | 118 (50.0) | 33 (47.8) | |
| Neutrophil | | | | 0.761 |
| ≤3.76 | 152 (49.8) | 116 (49.2) | 36 (52.2) | |
| ＞3.76 | 153 (50.2) | 120 (50.8) | 33 (47.8) | |
| Monocyte | | | | 0.505 |
| ≤0.41 | 155 (50.8) | 117 (49.6) | 38 (55.1) | |
| ＞0.41 | 150 (49.2) | 119 (50.4) | 31 (44.9) | |
| Hemoglobin | | | | 0.009 |
| ≤135.4 | 155 (50.8) | 130 (55.1) | 25 (36.2) | |
| ＞135.4 | 150 (49.2) | 106 (44.9) | 44 (63.8) | |
| Platelet | | | | 0.466 |
| ≤242 | 154 (50.5) | 116 (49.2) | 38 (55.1) | |
| ＞242 | 151 (49.5) | 120 (50.8) | 31 (44.9) | |
| Albumin* | - | - | - | - |
| Globulin | | | | 0.78 |
| ≤30 | 157 (51.5) | 123 (52.1) | 34 (49.3) | |
| ＞30 | 148 (48.5) | 113 (47.9) | 35 (50.7) | |
| NLR (median [IQR]) | 1.82 [1.45, 2.46] | 1.82 [1.45, 2.49] | 1.81 [1.45, 2.41] | 0.688 |
| LMR (median [IQR]) | 4.95 [3.75, 6.18] | 4.93 [3.53, 6.19] | 5.03 [4.23, 6.15] | 0.153 |
| Lymphocyte (median [IQR]) | 1.96 [1.61, 2.46] | 1.96 [1.58, 2.44] | 1.91 [1.71, 2.62] | 0.278 |
| Neutrophil (median [IQR]) | 3.77 [2.97, 4.72] | 3.78 [3.00, 4.70] | 3.73 [2.89, 4.85] | 0.855 |
| Monocyte (median [IQR]) | 0.41 [0.34, 0.52] | 0.42 [0.34, 0.51] | 0.40 [0.30, 0.53] | 0.518 |
| Hemoglobin (median [IQR]) | 135 [129, 142] | 134 [128, 141] | 138 [132, 144] | 0.011 |
| Platelet (median [IQR]) | 242 [211, 283] | 244 [210, 285] | 236 [213, 274] | 0.599 |

| | | | | |
|---|---|---|---|---|
| Albumin (median [IQR]) | 45 [43, 46] | 44 [42, 45] | 48 [47, 50] | <0.001 |
| Globulin (median [IQR]) | 30 [28, 33] | 30 [28, 33] | 30.8 [27, 33] | 0.753 |
| **Post–Neoadjuvant Chemotherapy** | | | | |
| Lymphocyte | | | | 0.235 |
| ≤1.54 | 154 (50.5) | 124 (52.5) | 30 (43.5) | |
| ＞1.54 | 151 (49.5) | 112 (47.5) | 39 (56.5) | |
| Neutrophil | | | | 0.287 |
| ≤3.75 | 153 (50.2) | 114 (48.3) | 39 (56.5) | |
| ＞3.75 | 152 (49.8) | 122 (51.7) | 30 (43.5) | |
| Monocyte | | | | 0.488 |
| ≤0.53 | 159 (52.1) | 120 (50.8) | 39 (56.5) | |
| ＞0.53 | 146 (47.9) | 116 (49.2) | 30 (43.5) | |
| Hemoglobin | | | | 0.394 |
| ≤117 | 153 (50.2) | 122 (51.7) | 31 (44.9) | |
| ＞117 | 152 (49.8) | 114 (48.3) | 38 (55.1) | |
| Platelet | | | | 0.975 |
| ≤271 | 153 (50.2) | 119 (50.4) | 34 (49.3) | |
| ＞271 | 152 (49.8) | 117 (49.6) | 35 (50.7) | |
| Albumin | | | | <0.001 |
| ≤46.3 | 256 (83.9) | 211 (89.4) | 45 (65.2) | |
| ＞46.3 | 49 (16.1) | 25 (10.6) | 24 (34.8) | |
| Globulin | | | | 0.45 |
| ≤28 | 158 (51.8) | 119 (50.4) | 39 (56.5) | |
| ＞28 | 147 (48.2) | 117 (49.6) | 30 (43.5) | |
| Lymphocyte (median [IQR]) | 1.54 [1.19, 1.87] | 1.49 [1.17, 1.86] | 1.61 [1.34, 1.91] | 0.171 |
| Neutrophil (median [IQR]) | 3.75 [2.81, 4.81] | 3.79 [2.83, 4.83] | 3.34 [2.67, 4.65] | 0.184 |
| Monocyte (median [IQR]) | 0.53 [0.42, 0.68] | 0.53 [0.42, 0.68] | 0.52 [0.42, 0.67] | 0.568 |
| Hemoglobin (median [IQR]) | 117 [110, 124] | 116 [109, 123] | 118 [111, 128] | 0.088 |
| Platelet (median [IQR]) | 271 [230, 335] | 270 [227, 333] | 272 [240, 347] | 0.529 |
| Albumin (median [IQR]) | 43.0 [41.0, 45.0] | 43. [40.9, 45.0] | 44.0 [42.0, 47.0] | <0.001 |
| Globulin (median [IQR]) | 28.0 [25.0, 31.0] | 28.0 [25.9, 31.0] | 27.0 [24.5, 31.0] | 0.217 |

*pre-neoadjuvant chemotherapy LMR and Alb were excluded from correlation analysis because that they were the compositions of SIS.

Abbreviation: SIS, systemic inflammation score; NLR, neutrophil to lymphocyte ratio; LMR, lymphocyte to monocyte ratio.
